# Clinical Characteristics and Outcomes of Gastric Cancer Patients Aged over 80 Years: A Retrospective Case-Control Study

**DOI:** 10.1371/journal.pone.0167615

**Published:** 2016-12-12

**Authors:** Hee Jung Park, Ji Yong Ahn, Hwoon-Yong Jung, Jeong Hoon Lee, Kee Wook Jung, Do Hoon Kim, Kee Don Choi, Ho June Song, Gin Hyug Lee, Jin-Ho Kim, Seungbong Han

**Affiliations:** 1 Department of Gastroenterology, University of Ulsan College of Medicine, Asan Medical Center, Asan Digestive Disease Research Institute, Seoul, Korea; 2 Department of Clinical Epidemiology and Biostatistics, University of Ulsan College of Medicine, Asan Medical Center, Seoul, Korea; Chang Gung Memorial Hospital Kaohsiung Branch, TAIWAN

## Abstract

**Background and Aims:**

The average human life expectancy is increasing worldwide, thus the proportion of elderly gastric cancer patients is also increasing. In this case-control study, we investigated the clinical and oncologic outcomes of gastric cancer in patients over 80 years old.

**Methods:**

From January 2004 to December 2010, 291 patients aged over 80 years old (case group) were diagnosed and treated with gastric cancer at Asan Medical Center, Seoul, Korea. From the same period, 291 patients aged 18 to 80 years old were selected as the control group. The clinical findings and clinical outcomes of gastric cancer were retrospectively reviewed and compared between the two groups.

**Results:**

There were significant differences in the overall 5-year survival rate between the case and control groups (30.9% vs. 73.8%, respectively; P<0.001). In patients who received the curative treatment, overall 3- and 5-year survival rates showed 74.3% and 57.9% in case group and 91.6% and 86.5% in the control group. When analysis was confined to resectable elderly patients with a favorable performance, the curative resection group showed significantly better overall 3- and 5-year survival rates than the conservative treatment group (73.7% and 58.8% vs. 29.8% and 0%, respectively).

**Conclusions:**

Although elderly gastric cancer patients show an advanced stage at diagnosis and poor prognosis compared with non-elderly patients, elderly patients with good performance could benefit from curative resection. Thus, the clinical decision whether to undergo curative resection or conservative management should be made on an individualized basis.

## Introduction

Gastric cancer is one of the most common malignancies in the world and is the second leading cause of cancer-related death worldwide [1]. Gastric cancer occurs predominantly in older age groups, with a peak incidence in the sixth decade of life [2]. However, the average human life expectancy is increasing worldwide, increasing the proportion of elderly gastric cancer patients. The clinicopathological features of gastric cancer in elderly patients are different from those of non-elderly patients. Features reported more frequently in elderly patients compared with non-elderly patients include a male predominance and significantly higher proportions of differentiated histologic tumor types, larger tumor sizes, and advanced TNM stages [3–5].

Treatments for elderly gastric cancer patients are controversial. Elderly patients have more comorbidities than non-elderly patients and surgery or conventional chemotherapy could be harmful rather than beneficial in some patients. Although several studies have analyzed the outcomes of elderly patients with gastric cancer [3,6,7]. those reports only included patients who underwent surgery and few reports describe the treatment outcomes of elderly gastric cancer patients in general.

There is no clear-cut definition for “elderly”. The average life expectancy at birth in many developed countries is over 80. In Japan, the average life expectancy now approaches 82 years [8], the highest among the world’s more developed countries, and is at least 79 years in several other developed countries [9]. In the case of Korea, the average life expectancy at birth was 81.4 years in 2012 [10]. Taking into consideration the aging population, in the present study, we defined our elderly group as patients aged over 80 years. We thus investigated the clinical and oncologic outcomes in elderly patients by this definition who were treated at the Asan Medical Center, Seoul, Korea via a retrospective case-control study.

## Patients and Methods

### Patients

**Fig 1:**
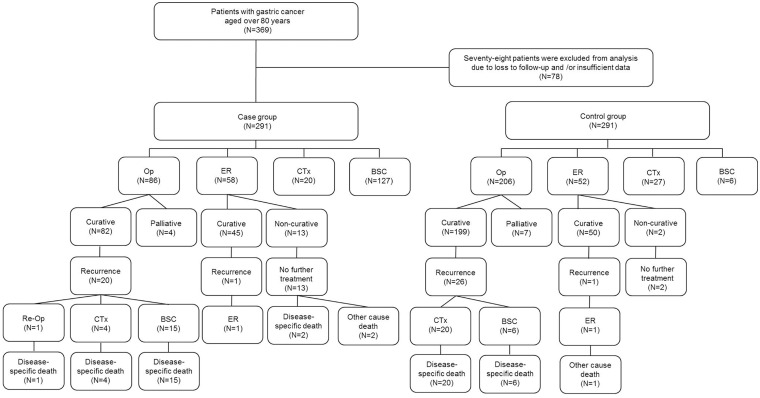
Flow chart of patients included in this study. A total of 291 patients aged over 80 years were included, and another 291 patients aged 80 years or younger were randomly selected as a control group. Op, operation; ER, endoscopic resection; CTx, chemotherapy; BSC, best supportive care.

From January 2004 to December 2010, 369 patients over 80 years old were diagnosed with gastric cancer at Asan Medical Center. Of these patients, 78 were excluded from this study due to insufficient data for analysis or loss to follow-up. Thus, 291 elderly patients were included in the analysis and defined as the case group (Fig 1). Among 27,457 patients aged 80 years or younger who were diagnosed with gastric cancer in the same period, a total of 291 patients were randomly selected as the control group for this case-control study.

In all cases, diagnosis was made based on the results of an upper gastrointestinal endoscopy and biopsy. Demographic data, clinical findings, histopathological parameters, and clinical outcomes were retrospectively reviewed using the electronic medical record database. The physical status of study patients was classified according to the American Society of Anesthesiologists (ASA) physical status classification [11]. Clinicopathological factors and treatment modalities were compared between the two groups and clinical outcomes were analyzed. The flow chart of enrolled patients is shown in Fig 1.

We obtained information on the status, including cause of death, of patients who were lost to follow-up by telephone survey under verbal consent. We also investigated any cause of death that we could not determine by telephone through the Office for National Statistics. This study was approved by the Institutional Review Board of Asan Medical Center (2012–5 0026).

### Definitions

Resectable gastric cancer was defined as the absence of distant metastasis or the invasion or encasement of major vascular structures such as the aorta, celiac axis, and splenic artery. Macroscopic types of gastric cancer were classified according to the classification of the Japanese Gastric Cancer Association [12] and the tumor degree of differentiation was classified as recommended by the World Health Organization [13]. Well- or moderately differentiated tubular cancer was classified as a differentiated type, whereas poorly differentiated adenocarcinoma, signet ring cell carcinoma, and mucinous carcinoma were classified as the undifferentiated type [12]. In addition, the histological subtypes of gastric cancer were classified according to Lauren’s criteria as intestinal and diffuse types [14].

In surgical cases, resection was considered complete when microscopic examination revealed a negative resection margin and no evidence of lymphovascular or perineural invasion. Resection was considered incomplete when microscopic examination revealed a positive tumor margin (R1) or residual gross disease (R2). Endoscopic resections were deemed curative when the lateral margins were free of tumor for more than 2 mm and the vertical margins were more than 0.5 mm on histologic examination and there was no evidence of submucosal invasion or lymphatic or vascular involvement. A positive family history of gastric cancer was defined as having one or more first- or second-degree relatives with a history of gastric cancer diagnosed at any age.

### Treatment protocols for gastric cancer at our institution

Endoscopic resection and surgery were performed in patients with resectable gastric cancer, whereas patients with unresectable gastric cancer were treated with chemotherapy or conservative management. The decision to perform curative or conservative treatment was at the discretion of the individual physician upon consideration of the disease state and patients’ performance status.

Endoscopic resection was performed in selected cases of early gastric cancer when there was no evidence of lymph node metastasis and the indication criteria were met, including (1) mucosal cancer of any size without ulceration and (2) mucosal cancer with ulcerations sized ≤ 30 mm or submucosal cancer less than 30 mm and confined to the upper 0.5 mm of the submucosa without lymphovascular invasion [15,16]. In endoscopic resection, the typical sequential procedure included marking, mucosal incision, and submucosal dissection with simultaneous hemostasis. After making several marking dots outside the lesion, saline containing epinephrine and indigo carmine was injected into the submucosal layer. A circumferential incision was made and submucosal dissection was performed to completely remove the lesion.

Gastrectomy was performed for patients who did not meet the criteria for endoscopic resection. Resection of both the tumor tissue plus appropriate lymph nodes was performed in cases of lymph node involvement or suspected lymph node metastasis during preoperative staging. Either subtotal or total gastrectomy was performed, depending upon the tumor location. Laparoscopic-assisted gastrectomy has been performed in our institute since 2004.

### Statistical analysis

Baseline continuous and categorical variables are presented as mean with standard deviation and number with percentage, respectively. Group comparisons of continuous variables were performed using Student’s *t*-tests, whereas categorical variables were compared with Fisher’s exact test or Pearson’s chi-square test. The Mann–Whitney *U* test was used for nonparametric analysis. Patients’ survival duration and differences in survival were estimated using the Kaplan–Meier method and log-rank tests among different patient subgroups. Factors possibly affecting death were analyzed by univariate and multivariate Cox proportional hazards regression analysis. Variables that were deemed of potential importance to the univariate analysis (*P<*0.1) were included in the multivariate analysis. The final models were determined by backward variable selection, a method whereby the least significant variables are removed one at a time. Results for significant prognostic factors were expressed as the hazard ratio for each category and its 95% confidence interval. All *P* values were two-sided, and *P*<0.05 was considered statistically significant. Statistical analyses were performed using SPSS for Windows version 19 (SPSS, Inc., Chicago, IL).

## Results

The mean age of all patients was 70.1±15.8 years and there were 419 (72.0%) male patients. The mean age of the case group was 83.6±2.7 years and there were 205 male patients (70.4%). The mean age of the control group was 56.6±11.2 years and there were 214 male patients (73.5%).

### Baseline characteristics of the case and control groups

Table 1 summarizes the clinical and histopathologic features of the two study groups. The case group showed a tendency for higher ASA physical status scores, a macroscopic finding of more advanced gastric cancer (62.2% vs. 38.1%; *P<*0.001), a histologic finding of more differentiated cancer (58.1% vs. 48.1%; *P =* 0.016), a more advanced stage (stage I: 40.2% vs. 68.0%; stage II: 13.1% vs. 6.9%; stage III: 21.6% vs. 12.7%; and stage IV: 25.1% vs. 12.4%; *P<*0.001), and less resectability (74.9% vs. 87.6%; *P<*0.001) than the control group. The most common symptom in both groups was epigastric pain (37.4% vs. 56.3%), followed in descending order by dyspepsia (22.6% vs. 20.0%), bleeding or anemia (15.4% vs. 9.6%), nausea or vomiting (8.6% vs. 3.7%), weight loss (7.7% vs. 8.9%), poor oral intake (4.1% vs. 0.7%), and dysphagia (4.1% vs. 0.7%). When symptoms were further classified as alarm symptoms according to the presence of one or more specific symptoms—bleeding or anemia, weight loss, dysphagia, persistent vomiting, and rapidly progressive symptoms—there was a marginally significant difference between the two groups, with a tendency for more alarm symptoms in the case group (28.7% vs. 19.3%; *P* = 0.051). The symptom duration before diagnosis was shorter in the case group than the control group (2.5±2.7 months vs. 3.2±3.2 months; *P* = 0.049).

**Table 1:** Baseline clinical and pathologic characteristics of gastric cancer in the case and control groups.

Variable	Case group (*N* = 291)	Control group (*N* = 291)	*P*
**Age (years)**	83.6±2.7	56.6±11.2	<0.001
**Sex**			0.406
**Male**	205 (70.4)	214 (73.5)	
**Female**	86 (29.6)	77 (26.5)	
**Family history**	26 (9.3)	48 (16.8)	0.008
**BMI (kg/m**^**2**^**)**	22.14±3.48	23.35±3.09	<0.001
**Presence of symptoms**	195 (67.0)	135 (46.4)	<0.001
**Underlying comorbidities**	188 (64.6)	108 (37.1)	<0.001
**ASA physical status**^a^	2.3±0.7	1.9±0.7	<0.001
**1**	33 (11.3)	89 (30.6)	
**2**	147 (50.5)	152 (52.2)	
**3**	105 (36.1)	49 (16.8)	
**4**	6 (2.1)	1 (0.3)	
***Helicobacter pylori* infection**	69/104 (66.3)	87/126 (69.0)	0.662
**Tumor size (cm)**	4.5±2.4	4.0±3.0	0.013
**Macroscopic type**			< 0.001
**EGC**	110 (37.8)	180 (61.9)	
**AGC**	181 (62.2)	111 (38.1)	
**Tumor location**			0.302
**Lower**	164 (56.4)	146 (50.2)	
**Middle**	88 (30.2)	106 (36.4)	
**Upper**	38 (13.1)	38 (13.1)	
**Diffuse**	1 (0.3)	1 (0.3)	
**Differentiation**			0.016
**Differentiated**^b^	169 (58.1)	140 (48.1)	
**Undifferentiated**^c^	122 (41.9)	151 (51.9)	
**Lauren’s classification**			<0.001
**Diffuse**	16 (13.1)	80 (34.9)	
**Intestinal**	99 (77.0)	124 (54.1)	
**Mixed**	12 (9.8)	25 (10.9)	
**Clinical stage**^d^			<0.001
**I**	117 (40.2)	198 (68.0)	
**II**	38 (13.1)	20 (6.9)	
**III**	63 (21.6)	37 (12.7)	
**IV**	73 (25.1)	36 (12.4)	
**Resectability**			<0.001
**Resectable**	218 (74.9)	255 (87.6)	
**Unresectable**	73 (25.1)	36 (12.4)	

Data represent number of patients (%) or mean. BMI, body mass index; CEA, carcinoembryonic antigen; CA 72–4; cancer antigen 72–4; EGC, early gastric cancer; AGC, advanced gastric cancer.

^a^ASA physical status refers to the physical status classification of the American Society of Anesthesiologists.

^b^Differentiated carcinomas include well- or moderately differentiated, tubular or papillary adenocarcinomas.

^c^Undifferentiated carcinomas include poorly differentiated adenocarcinomas, signet ring cell carcinomas, and mucinous carcinomas.

^d^Clinical stage was established according to the guidelines of the 7^th^ American Joint Committee on Cancer.

The case group had more underlying comorbidities than the control group. Of comorbidities, cardiovascular disease (*n* = 158, 54.3%), including hypertension, atrial fibrillation, ischemic heart disease, heart failure, and valvular heart disease, was the most common comorbidity in the case group. Other prevalent comorbidities in the case group included, in descending order, diabetes mellitus (*n* = 43, 14.8%), cerebrovascular accident (*n* = 18, 6.2%), pulmonary disease including chronic obstructive pulmonary disease, restrictive pulmonary disease, and uncontrolled asthma (*n* = 18, 6.2%), other cancers (*n* = 15, 5.2%), and chronic kidney disease (*n* = 1, 0.3%). The control group also showed a similar order of prevalent comorbidities: cardiovascular disease (*n* = 80, 27.5%), diabetes mellitus (*n* = 30, 10.3%), other cancers (*n* = 9, 3.1%), cerebrovascular accident (*n* = 8, 2.7%), pulmonary disease (*n* = 5, 1.7%), chronic kidney disease (*n* = 3, 1.0%), and liver cirrhosis (*n* = 1, 0.3%).

### Overall oncologic outcomes of the case and control groups

**Fig 2:**
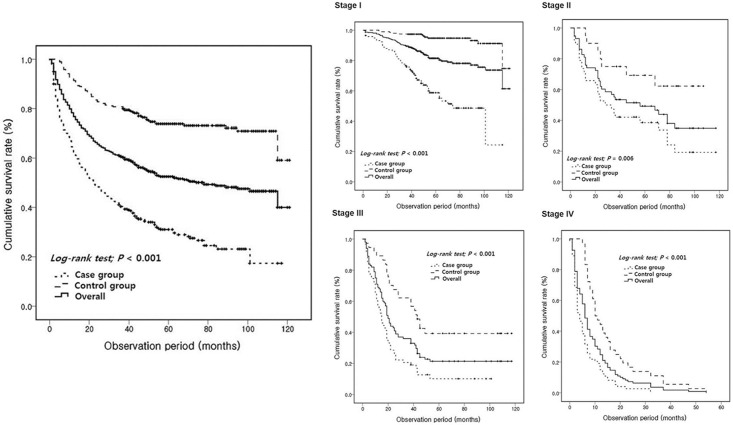
Cumulative survival rates between case and control groups. **(A)** Kaplan–Meier survival curve showing a significant difference in overall survival between the control and case groups (*P*<0.001). **(B)** Kaplan–Meier estimates of overall survival rates in patients of the control and case groups according to the tumor stage. The case group showed significantly lower survival rates at each stage than the control group (stage I, *P*<0.001; stage II, *P* = 0.006; stage III, *P*<0.001; and stage IV, *P*<0.001).

The median follow-up period of all patients was 42 months (interquartile range [IQR] 14–70 months) and the mortality rate was 48.6%. The median follow-up period of the case group was 22 months (IQR 6–51), which was significantly lower than that of the control group (56 months, IQR 40–89; *P<*0.001; Table 2). The overall 3- and 5-year survival rates in all patients were 60.9% and 52.5%, respectively. The 3- and 5-year survival rates of the case group were 40.4% and 30.9%, respectively, whereas those of the control group were 80.8% and 73.8%, respectively, which were significantly different (Fig 2A). Survival analyses of the case and control groups in each stage showed a similar result, with a lower survival of the case group than the control group (Fig 2B). In the case group, 205 patients (70.4%) died during the follow-up period. Of these, 156 patients died of disease-specific causes; the other 49 patients died of disease-unrelated causes (13 patients with cancers of other origins, 13 patients with pneumonia, 7 patients with systemic infection, 5 patients with cardiovascular disease including ischemic heart disease, arrhythmia, and heart failure, 4 patients with asphyxia, 3 patients with cerebral hemorrhage, 2 patients with renal failure, and 2 patients with traffic accident). In the control group, 78 patients (26.8%) died during the follow-up period. Of these, 62 patients died of disease-specific causes; the other 16 patients died of disease-unrelated causes (10 patients with cancers of other origins, 3 patients with pneumonia, 1 patient with systemic infection, 1 patient with cardiovascular disease, and 1 patient with renal failure). When survival analysis was confined to disease-specific causes, there was no significant difference in mortality between the case and control groups (76.1% vs. 79.5% respectively; *P* = 0.545).

**Table 2:** Oncologic outcomes of gastric cancer in the case and control groups.

Variable	Case group(*N* = 291)	Control group(*N* = 291)	*P*
**Treatment modality**			<0.001
**Surgical resection**	86 (29.6)	206 (70.8)	
**Endoscopic resection**	58 (19.9)	52 (17.9)	
**Chemotherapy**	20 (6.9)	27 (9.3)	
**Conservative treatment**	127 (43.6)	6 (2.1)	
**Median follow-up period**	22 (6–51)	56 (40–89)	<0.001
**Stage 1**	48 (36–64)	65 (49–94)	<0.001
**Stage 2**	31 (9–66)	64 (28–87)	0.020
**Stage 3**	15 (7–26)	43 (21–70)	<0.001
**Stage 4**	4 (2–8)	11 (7–20)	<0.001
**Overall 5-year survival rate (%)**	30.9	73.8	<0.001
**Mortality rate**	205 (70.4)	78 (26.8)	<0.001
**Disease-specific mortality**	156 (76.1)	62 (79.5)	0.545

Data represent the number of patients (%) and the median follow-up period is presented with the median and interquartile range (IQR).

We analyzed the comparison between case and control groups confined to patients who received the curative treatment with surgery or endoscopic resection in resectable gastric cancers. Surgery and endoscopic resection were performed in 82 and 58 cases among case group, and 199 and 52 cases among control group, respectably. In the case group, 64 patients (45.7%) died during the follow-up period and 25 (39.1%) died of disease-specific causes of them. Thirty-nine patients (15.5%) were died and 24 (61.5%) died of disease-specific causes of them. The overall 3- and 5-year survival rate was 74.3% and 57.9% in the case group and 91.6% and 86.5% in the control group (Table 3).

**Table 3:** Oncologic outcomes of curative treated gastric cancer in the case and control groups.

Variable	Case group (N = 140)	Control group (N = 251)	*P*
**Age (years)**	83.2	56.9	<0.001
**Sex**			0.808
**Male**	106 (75.7)	186 (74.1)	
**Female**	34 (24.3)	65 (25.9)	
**Presence of symptoms**	81 (57.9)	108 (43.0)	0.006
**Tumor size (cm)**	41.2	35.2	0.039
**Macroscopic type**			0.033
**EGC**	85 (60.7)	179 (71.3)	
**AGC**	55 (39.3)	72 (28.7)	
**Clinical stage**^a^			0.013
**I**	91 (65.0)	196 (78.1)	
**II**	21 (15.0)	19 (7.6)	
**III**	28 (20.0)	36 (14.3)	
**Overall 5-year survival rate**	57.9	86.5	<0.001
**Death**	64 (45.7)	39 (15.5)	<0.001
**Disease-specific mortality**	25 (39.1)	24 (61.5)	<0.001

Data represent number of patients (%) or mean. EGC, early gastric cancer; AGC, advanced gastric cancer.

^a^Clinical stage was established according to the guidelines of the 7^th^ American Joint Committee on Cancer.

### Oncologic outcomes of the case and control groups according to the treatment modality

Oncologic outcomes according to the treatment modality are shown in Table 4. There was a significant difference in treatment modalities between the case and control groups (*P*<0.001). In patients with surgical resection, there were significant differences between the two groups in the overall 5-year survival rate and overall mortality (*P*<0.001 and *P*<0.001, respectively). Patients who underwent endoscopic resection also showed significant differences in the overall 5-year survival rate and overall mortality between the two groups (*P* = 0.022 and *P* = 0.011, respectively). Of the 86 patients who underwent surgery in the case group, there were 82 cases of curative resection and 4 of palliative resection. Of the 82 curative surgical resections, 31 patients (37.8%) showed complete resection, R1 resection was performed in 51 patients (62.2%), and recurrence occurred in 20 patients. Of the 58 patients who underwent endoscopic resection in the case group, there were 45 cases of curative resection and 13 of non-curative resection. There was one case of recurrence among the patients who underwent curative endoscopic resection. Among 31 patients with R0 resection, two patients died due to perioperative complication. One died due to anastomosis site leakage which was happen 20 days after operation and other died due to post-operative bleeding 15 days after operation.

**Table 4:** Oncologic outcomes according to the treatment modality.

Treatment modality	Case group (*N* = 291)	Control group (*N* = 291)	*P*
**Surgical resection**	86 (29.6)	206 (70.8)	
**ASA physical status**^a^	2.1±0.8	1.9±0.7	0.013
**1**	17 (19.8)	59 (28.6)	
**2**	40 (46.5)	112 (54.4)	
**3**	29 (33.7)	34 (16.5)	
**4**	0 (0)	1 (0.5)	
**Overall 5-year survival rate (%)**	45.2	85.3	<0.001
**Death**	48 (55.8)	39 (18.9)	<0.001
**Disease-specific death**	28/48 (58.3)	31/39 (79.5)	0.036
**Endoscopic resection**	58 (19.9)	52 (17.9)	
**ASA physical status**	2.2±0.7	1.8±0.7	0.003
**1**	8 (13.8)	18 (34.6)	
**2**	32 (55.2)	27 (51.9)	
**3**	18 (31.0)	7 (13.5)	
**Overall 5-year survival rate (%)**	55.1	78.7	0.022
**Death**	20 (34.5)	7 (13.5)	0.011
**Disease-specific death**	1/20 (5.0)	0/7 (0)	>0.999
**Chemotherapy**	20 (6.9)	27 (9.3)	
**ASA physical status**	2.3±0.6	1.8±0.8	0.024
**1**	1 (5.0)	11 (40.7)	
**2**	13 (65.0)	11 (40.7)	
**3**	6 (30.0)	5 (18.5)	
**Overall 5-year survival rate (%)**	5.0	0	0.358
**Death**	19 (95.0)	27 (100.0)	0.426
**Disease-specific death**	19/19	27/27	
**Conservative treatment**	127 (43.6)	6 (2.1)	
**ASA physical status**	2.5±0.7	2.3±0.8	0.684
**1**	7 (5.5)	1 (16.7)	
**2**	62 (48.8)	2 (33.3)	
**3**	52 (40.9)	3 (50.0)	
**4**	6 (4.7)	0 (0)	
**Overall 5-year survival rate (%)**	8.2	0	0.302
**Death**	118 (92.9)	5 (83.3)	0.380
**Disease-specific death**	108/118 (90.5)	4/5 (80.0)	0.379

Data represent the number of patients (%) or mean.

^a^ASA physical status refers to the physical status classification of the American Society of Anesthesiologists.

Of 127 patients who were managed with conservative treatment in the case group, 55 had stage 4 and the other 72 had a resectable stage. The reasons for conservative treatment in resectable patients were inoperability due to poor performance status in 38 patients (52.8%), refusal of surgery or endoscopic resection in 27 patients (37.5%), and other comorbidities such as lung cancer, colon cancer, or coronary artery disease in 7 patients (9.7%). Curative-intent treatment comprising surgical and endoscopic resection was more commonly performed in the control group (49.5% vs. 88.7%, *P<*0.001).

**Fig 3:**
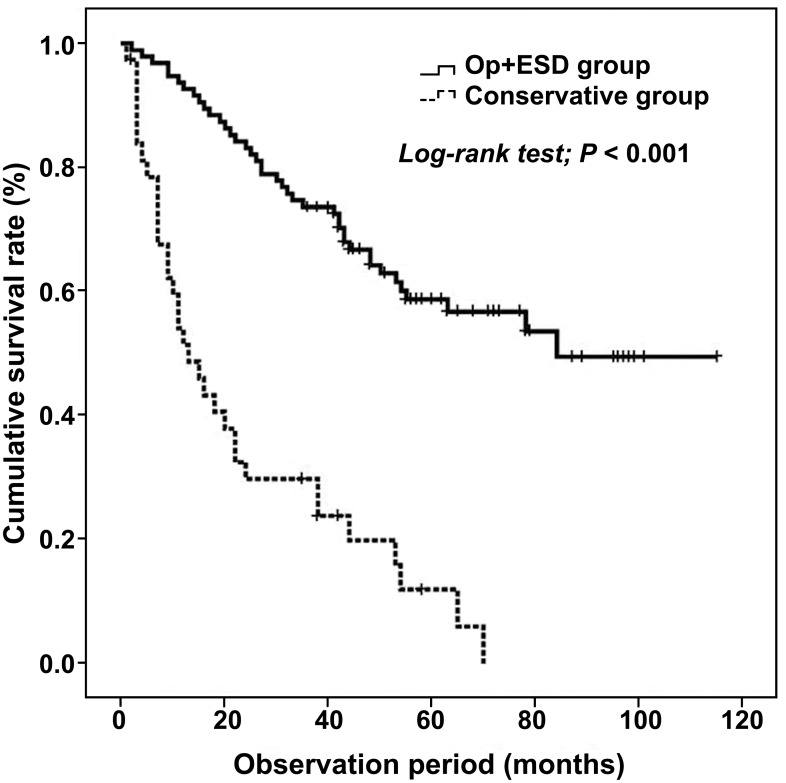
Kaplan–Meier estimates of survival rates in resectable elderly patients with a favorable performance (ASA score 1 or 2) according to the treatment modality. The curative resection group showed better survival than the conservative treatment group (*P*<0.001).

When analysis was confined to resectable elderly patients with a favorable performance (ASA score 1 or 2), curative resection (endoscopic or surgical resection) was performed in 95 patients and conservative treatment was performed in 38 patients. In curative resection group, clinical stage I, II, and III were 65 (68.4%), 11 (11.6%), 19 (20.0%) cases respectably and clinical stage I, II, and III were 12 (31.6%), 10 (26.3%), 16 (42.1%) cases respectably in conservative treatment group. The overall 3-year survival rate was 73.7% in the curative resection group and 29.8% in the conservative treatment group. The overall 5-year survival rate was 58.8% in the curative resection group and 0% in the conservative treatment group. The median follow-up duration was 48 months and 13 months, respectively. The curative resection group showed significantly better survival than the conservative treatment group (Fig 3).

The other analysis of clinical outcomes about the surgery and conservative management in early stage (clinical stage I and II) of case group with good performance (ASA score 1 or 2) showed that overall 5-year survival rate was 50% in surgery group and 9.1% in conservative management group (*P*<0.001, S1 Table).

### Univariate and multivariate analysis of the potential risk factors for death in the case group

Significant prognostic factors for lower survival in univariate analysis included the presence of symptoms, a lower BMI, the ASA physical status score, a larger tumor size, a macroscopic type of advanced gastric cancer, an undifferentiated tumor type, and an advanced TNM stage (Table 4). By multivariate analysis with backward elimination, a tumor size (hazard ratio [HR] 1.008, 95% CI 1.000–1.016, *P =* 0.047), advanced TNM stage (stage 3: HR 3.212, 95% CI 1.879–5.492, *P<*0.001; stage 4: HR 8.249, 95% CI 4.661–14.597, *P<*0.001), and conservative treatement (HR 3.574, 95% CI 2.372–5.386, *P<*0.001) were found to be independent prognostic predictors of poorer survival. (Table 5).

**Table 5:** Univariate and multivariate analyses of prognostic factors of overall mortality in gastric cancer patients aged over 80 years.

Factors	Univariate analysis			Multivariate analysis
	HR (95% CI)	*P*	HR (95% CI)	*P*
**Age**	1.039 (0.993–1.087)	0.095	1.004 (0.947–1.063)	0.902
**Sex**				
**Female**	1.232 (0.919–1.651)	0.164		
**Family history**	1.070 (0.673–1.701)	0.774		
**Presence of symptoms**	2.109 (1.537–2.894)	<0.001	0.793 (0.530–1.185)	0.257
**BMI**	0.924 (0.883–0.967)	0.001	1.005 (0.958–1.055)	0.835
**ASA physical status score**	1.326 (1.088–1.616)	0.005	1.158 (0.917–1.462)	0.217
**Tumor size**	1.015 (1.010–1.020)	<0.001	1.008 (1.000–1.016)	0.047
**Macroscopic type**				
**EGC**				
**AGC**	3.452 (2.512–4.745)	<0.001	0.660 (0.349–1.249)	0.202
**Tumor location**				
**Lower**				
**Middle**	0.773 (0.562–1.063)	0.114		
**Upper**	1.236 (0.828–1.847)	0.300		
**Differentiation**				
**Differentiated**^a^				
**Undifferentiated**^b^	1.626 (1.233–2.145)	0.001	1.099 (0.792–1.524)	0.573
**TNM stage**				
**Stage 1**				
**Stage 2**	2.151 (1.345–3.440)	0.001	1.770 (0.993–3.155)	0.053
**Stage 3**	4.406 (2.971–6.534)	<0.001	3.212 (1.879–5.492)	<0.001
**Stage 4**	15.642 (10.300–23.755)	<0.001	8.249 (4.661–14.597)	<0.001
**Treatment modality**				
**Surgical resection**				
**Endoscopic resection**	0.528 (0.313–0.890)	0.016	1.212 (0.642–2.290_	0.554
**Chemotherapy**	3.350 (1.959–5.728)	<0.001	1.569 (0.857–2.872)	0.144
**Conservative treatment**	4.009 (2.836)	<0.001	3.574 (2.372–5.386)	<0.001

BMI, body mass index; CEA, carcinoembryonic antigen; CA 72–4; cancer antigen 72–4; EGC, early gastric cancer; AGC, advanced gastric cancer.

^a^Differentiated carcinomas include well- or moderately differentiated, tubular or papillary adenocarcinomas.

^b^Undifferentiated carcinomas include poorly differentiated adenocarcinomas, signet ring cell carcinomas, and mucinous carcinomas.

## Discussion

In the present case-control study, we investigated the clinicopathological features, treatment outcomes, and survival of gastric cancer in elderly patients. Our results showed that gastric cancer in elderly patients had different clinicopathological characteristics than in non-elderly patients. Regarding overall oncologic outcomes, the 3- and 5-year survival rates of the case group were worse than those of the control group with an advanced stage cancer at diagnosis. Among resectable elderly patients with a favorable performance (ASA score 1 or 2), the curative resection group showed significantly better survival than the conservative treatment group. Thus, good prognosis can be expected following early detection of gastric cancer and proper treatment in patients with good performance.

It has been suggested that gastric cancer in elderly patients has distinguishing clinical and histopathologic profiles [4,5,17]. Our results showed different characteristics of elderly patients compared with non-elderly patients, as follows: a less frequent positive family history, a lower BMI, more presence of symptoms, more underlying comorbidities, lower levels of hemoglobin, cholesterol, and albumin, a larger tumor size, a more macroscopic type of advanced gastric cancer, a more differentiated tumor type, a more advanced TNM stage, and lower resectability. Several studies suggested that gastric cancer in the elderly progresses to undifferentiated tumor after development as a differentiated tumor, whereas in young patients, gastric cancer manifests as an undifferentiated tumor at the initial stage [5]. The predominance of an intestinal type of cancer in the elderly could be explained by its mechanism of occurrence, which is associated with environmental factors such as obesity, dietary factors, and cigarette smoking, as well as with infection with *Helicobacter pylori* [18–20].

The more commonly observed presence of symptoms in the elderly is also considered to be one of the manifestations of an advanced stage cancer. One study has shown that gastric cancer incidence in any individual without alarm symptoms is very low [21]. However, in a prospective study that investigated the detection rate of gastric cancer in dyspeptic patients, there was an increased detection rate of gastric cancer, followed by an increased operability, showing a potential risk of dyspeptic symptoms, even though dyspepsia is not an alarm symptom [22]. In our study patients, the most commonly presented symptoms were epigastric pain, followed by dyspepsia. The proportions of alarm symptoms among symptomatic patients were 28.7% and 19.3% in the case and control groups, respectively, representing more symptoms not included as alarm symptoms. The reason for the more advanced stage of cancer at diagnosis in the elderly is unclear and there are no known explanations. It is uncertain whether this phenomenon is a unique characteristic of elderly gastric cancer in itself or a coincidence of detection at a later stage affected by differences in medical surveillance. When analysis was confined to symptomatic patients, our results showed a lower clinical stage and favorable outcome in patients without symptoms at diagnosis than with symptoms in the case group.

Regarding treatment modalities, elderly patients showed a trend toward less invasive treatments than non-elderly patients. Curative resection, including endoscopic or surgical resection, was more frequently performed in the case group than the control group in both the overall and resectable population analyses. The reason for conservative treatment of resectable patients was mainly inoperability (52.8%) due to poor performance status. There was a significant difference in overall survival between the two age groups, although there was no difference in cancer-specific survival. In the elderly group, cancer-unrelated deaths were more common than in the non-elderly group, in line with previous studies [3,23]. These findings could be a sign that there are no specific characteristics that affect poorer survival in elderly gastric cancer patients.

In a retrospective study involving patients with gastric cancer aged 85 years or older [24], patients who underwent curative resection had a significantly better survival than those managed with conservative treatment. Our study had a similar result. In the case group, patients with resectable disease showed better survival in the curative resection (endoscopic or surgical resection) group than in the conservative treatment group. In subgroup analysis, resectable elderly patients with a favorable performance (ASA score 1 or 2) in the curative resection (endoscopic or surgical resection) group showed significantly better survival than in the conservative treatment group. Therefore, considering this evidence, age should not be an issue anymore for treatment decisions for gastric cancer patients in this aging society. Resectable elderly patients with good performance status could benefit from curative resection of gastric cancer. However, before making decisions, further consideration should be given to the experience and ability of the hospital for surgery and postoperative management.

As a retrospective single-center study, this study has limitations inherent to any retrospective and single-center analysis. However, the main strength of this study—sufficient cases of elderly gastric cancer patients with accompanying randomly selected controls—could overcome these limitations and show a well-powered evaluation of subpopulations with more accurate analysis of interactions.

In conclusion, our present data show that gastric cancer in elderly patients aged over 80 years has distinctive features, such as an advanced stage at diagnosis and poor prognosis, compared with non-elderly patients. In analysis of the case group, curative resection showed a favorable outcome in resectable elderly patients with good performance status. Although elderly patients showed an advanced stage at diagnosis and poor prognosis compared with non-elderly patients, elderly patients with good performance could benefit from curative resection of gastric cancer. Thus, the clinical decision whether to undergo curative resection or conservative management should be made via an individualized approach.

## Supporting Information

S1 Table Clinical outcomes of surgery compare to the conservative management in early stage of case group with good performance. (DOCX)
