# Supplementary material for: Clinical Characteristics and Outcomes of Gastric Cancer Patients Aged over 80 Years: A Retrospective Case-Control Study
Source: PLoS One. 2016 Dec 12;11(12):e0167615. doi: 10.1371/journal.pone.0167615 (PMC5152853; doi:10.1371/journal.pone.0167615)
Supplement: S1 Table — (DOCX) [file pone.0167615.s001.docx]

**S1 Table** **Clinical outcomes of surgery compare to the conservative management in early stage of case group with good performance**

| **Variable** | **Surgery (N=36)** | **Conservative management (N=22)** | ***P*** |
| --- | --- | --- | --- |
| **Age (years)** | 82.4 | 84.6 | 0.012 |
| **Sex** |  |  | 0.129 |
| **Male** | 29 (80.6) | 13 (59.1) |  |
| **Female** | 7 (19.4) | 9 (40.9) |  |
| **Presence of symptoms** | 23 (63.96) | 13 (59.1) | 0.384 |
| **Tumor size (cm)** | 44.9 | 36.1 | 0.120 |
| **Macroscopic type** |  |  | 0.427 |
| **EGC** | 19 (52.3) | 9 (40.9) |  |
| **AGC** | 17 (47.7) | 13 (59.1) |  |
| **Clinical stage^a^** |  |  | 0.206 |
| **Ia** | 15 (41.7) | 9 (40.9) |  |
| **Ib** | 10 (27.8) | 3 (13.6) |  |
| **IIa** | 7 (19.4) | 3 (13.6) |  |
| **IIb** | 4 (11.1) | 7 (31.9) |  |
| **Overall 5-year survival rate** | 50 | 9.1 | <0.001 |
| **Death** | 13 (36.1) | 18 (81.8) | <0.001 |

Data represent number of patients (%) or mean.

EGC, early gastric cancer; AGC, advanced gastric cancer.

^a^Clinical stage was established according to the guidelines of the 7^th^ American Joint Committee on Cancer.
